# Role of Confocal Laser Endomicroscopy in Detection of Residual Barrett's Esophagus after Radiofrequency Ablation

**DOI:** 10.1155/2011/593923

**Published:** 2011-10-19

**Authors:** Giorgio Diamantis, Paolo Bocus, Stefano Realdon, Giorgio Battaglia

**Affiliations:** ^1^Veneto Oncological Institute (IOV), IRCCS, 35128 Padua, Italy; ^2^Department of Surgical and Gastroenterological Sciences, University of Padua, 35128 Padua, Italy

## Abstract

Endoscopic endoluminal radiofrequency ablation (RFA) is a novel and promising modality for Barrett's esophagus (BE) treatment. Actually the only surveillance method after the ablation treatment is random biopsies throughout the whole treated area. Confocal laser endomicroscopy (CLE) is a new endoscopic imaging tool that permits high-resolution microscopic examination of the gastrointestinal tract. The technology has garnered increasing attention because of its ability to provide real-time “optical” biopsy specimens, with a very high sensitivity and specificity. This paper summarize the potential application of CLE in the surveillance of the reepithelialization of BE, after endoscopic RFA.

## 1. Introduction

CLE is a new endoscopic technique, which allows surface in vivo microscopic analysis during ongoing endoscopy, using systemically or topically administered fluorescent agents. CLE uses a single-line laser with a wavelength of 488 nm to generate optical histologic slices of 7 *μ*m [1]. It allows targeted biopsies to be taken, potentially improving the diagnostic rate in certain gastrointestinal diseases. The technology has garnered increasing attention because of its ability to provide real-time “optical” biopsy specimens, with a very high sensitivity and specificity [2]. Worldwide experience with CLE for upper gastrointestinal malignant and premalignant lesions is still limited. Potential clinical applications are presented, including diagnosis of NERD, BE [3, 4], early squamous cell carcinoma in the esophagus [5], atrophic gastritis, gastric intestinal metaplasia, dysplasia and gastric cancer [6], celiac disease [7], ulcerative colitis [8], and colorectal cancer [9].

The incidence of esophageal adenocarcinoma has risen steadily over the past 10 years. Patients with BE are at increased risk for the development of adenocarcinoma. The field of BE ablation has advanced dramatically in recent years. Endoscopic ablation is now viewed as a legitimate first-line treatment option for healthy patients with intestinal metaplasia (IM), low-/high-grade dysplasia and, in some cases, early adenocarcinoma, on the basis of ongoing research [10]. Recently there has been a growing literature related to the endoscopic ablation of BE using RFA [11, 12]. 

This case highlights another potential application of CLE: the BE surface study after endoscopic endoluminal RFA.

## 2. Case Report

**Figure 1:**
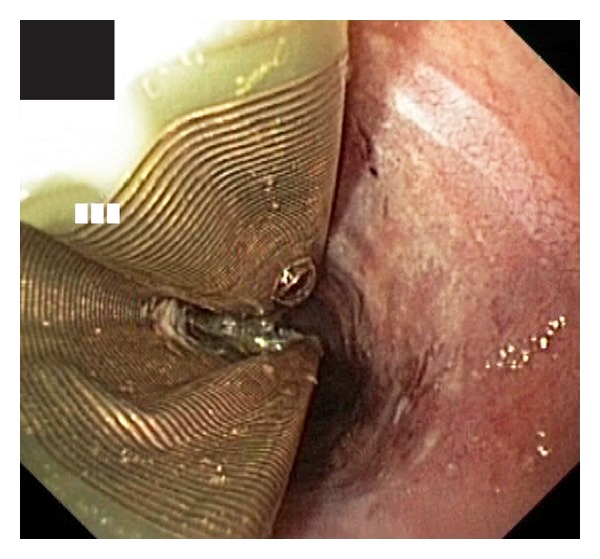
RFA catheter deflated inside the esophagus after the first ablating session. On the right is clearly visible the greyish ablated mucosa.

A 70-year-old man with a history of long-standing reflux, symptomatic GERD, histologic evidence of BE without dysplasia and a large hiatal hernia, was referred to our department for further management. After a Collis-Nissen surgical treatment, the endoscopic followup, over a 3-year period, revealed unchanged histological features: IM within the 8 cm segment of endoscopic BE (C7M8—Prague C & M Criteria), without dysplasia. These endoscopies were performed both with and without methylene blue stain, by using 4-quadrant biopsies every 2 cm throughout the BE (Seattle's biopsy protocol). After this surveillance period according with the patient and after an informed consent was obtained, we start to treat the BE by endoscopic endoluminal RFA (Barrx Halo 360 System). Under monitored anesthesia care and after the endoscopic esophageal landmarks were defined, the esophageal wall was sprayed with acetylcysteine 1% for the ablation procedure. The esophageal diameter was sized with a sizing catheter, passed endoscopically over a stiff guidewire, and then removed. An autosizing balloon of the ablation system was used to determine the diameter of the esophagus and allow good contact between the radiofrequency delivery system (balloon/electrodes) and the esophageal wall on one hand and not apply excessive pressure on the other. The RFA procedure started moving from distally to proximally, and the balloon was progressively repositioned allowing a very small overlap with the previous treated zone (Figure 1). The exudative material caused by the burn was scraped off the esophagus with aggressive washing and an endoscopic cap (used for mucosal resection). Because of the length, the ablation was repeated until the 2/3 of the BE (6 cm), were treated with radiofrequency energy, and carried out the treatment of the last 2 cm after two months (as suggested by the producers of the ablation system). The patient was discharged the same day with a prescription of esomeprazole 40 mg twice a day for the first month and 40 mg every day until the following examination. He was also instructed to eat only a soft diet for 2–4 days, use liquid acetaminophen for the eventual discomfort, and avoid aspirin and anti-inflammatory drugs for 7 days.

**Figure 2:**
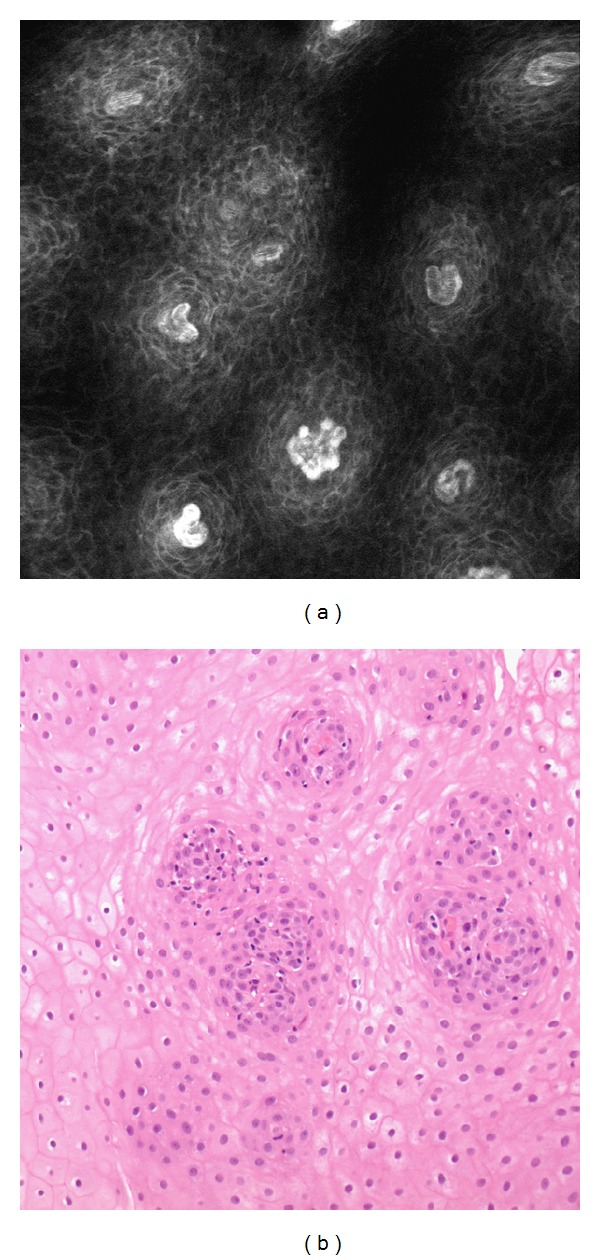
CLE image and histologic aspect (trasversal section) of the proximal treated esophageal area. Normal squamous esophagus showing individual epithelial cells and intrapapillary capillary loops.

**Figure 3:**
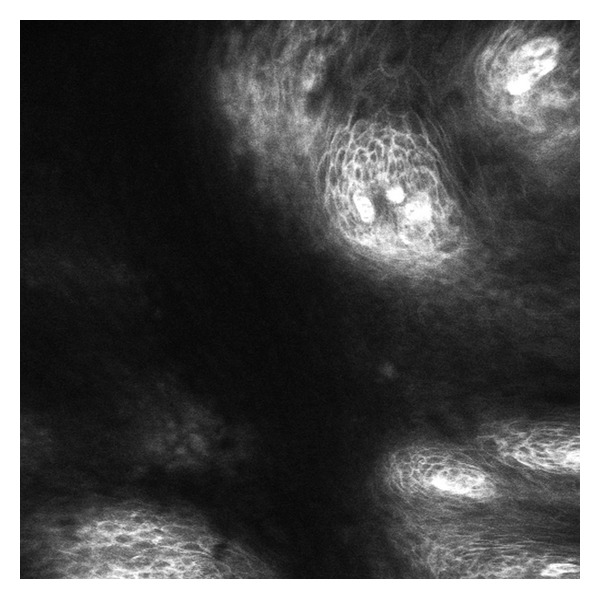
CLE image of the treated esophageal area, middle portion. Normal squamous esophagus showing individual epithelial cells and intrapapillary capillary loops.

**Figure 4:**
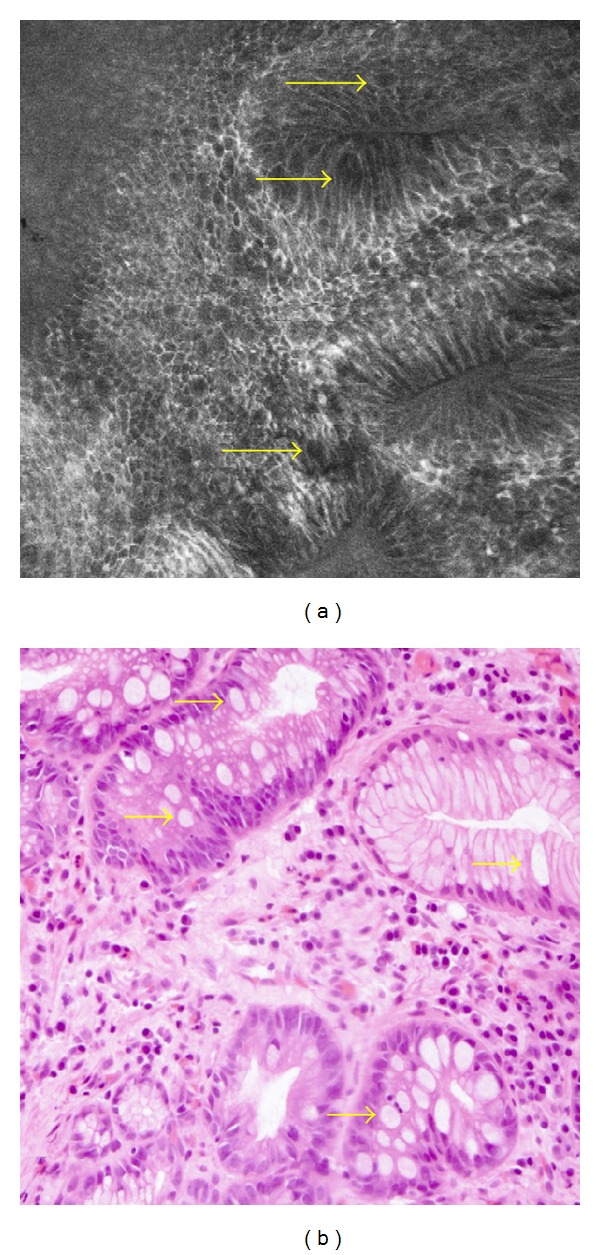
CLE image and histologic aspect (trasversal section) of the untreated esophageal zone: columnar lined epithelium with presence of focal mucin goblet cells (arrows), pathognomonic of gastric type mucosae with focal intestinal metaplasia.

After a month, we decided to control the treated area of the esophagus. Under monitored anesthesia care, the mucosa was initially examined with standard white-light endoscopy. Subsequently the patient was intubated with a confocal endomicroscope (EC-3870CIFK; Pentax, Tokyo, Japan). After an intravenous injection of 5 mL fluorescein sodium 10%, the 6 cm treated area was circumferentially imaged and stored digitally. We collect 78 images at a scan rate of 0,8 frames per second (1024 × 1024 pixels), using an optical slice thickness of 7 *μ*m, with lateral and axial resolution of 0.7 *μ*m. The microscopic field of view was 475 × 475 *μ*m with an infiltration depth of the blue laser light from the surface to 250 *μ*m. Endomicroscopy was performed in the whole treated esophagus, in the 4 quadrants mimicking the Seattle biopsy protocol [13], from the surface to the deeper portion. The site of interest was placed at the lower left corner of the CLE window and the distal tip of the endoscope in contact with the mucosa using blue laser as guide. The position of the focal plane within the specimen was adjusted using the buttons on the endoscope control panel. During the procedure, a gentle suction was used to stabilize the endomicroscope and minimize excessive movement, reducing motion artifacts. All images revealed a typical regular-appearing subepithelial capillary network. The capillary loops within the papillae were visible due to the high contrast of the fluorescein within the vascular structures, surrounded by normal epithelial cells (Figures 2 and 3). The same procedure was also performed over the proximal not treated surface. In that case, the CLE revealed regular-shape subepithelial capillaries underneath a columnar-lined epithelium with presence of focal dark mucin goblet cells in the upper parts of the mucosal layer (Figure 4). At that point, we concluded the examination performing 4-quadrant biopsies every 1 to 2 cm throughout both treated and not treated surface. The “optical biopsy” site was located 5 mm immediately to the left of the suction-marked area, obtained during the CLE examination. Histopathologic examination of the biopsy specimens confirmed the normal squamous epithelium over the whole treated esophageal area without any evidence of residual IM beneath the newly generated epithelium confirming the CLE diagnosis. The residual columnar epithelium was completely ablated with a single session of Halo 90°.

## 3. Discussion

Considering the fact that the RFA is a relatively new technique and there are no long-term studies showing an irreversible disappearance of the IM with complete healing of the esophagus, after many years, the only surveillance method is the random biopsies throughout the whole treated area. The longest follow-up study for patients, underwent endoscopic ablation of nondysplastic BE, is a prospective multicenter US trial published 1 year ago including 50 patients [12]. Of 1473 esophageal specimens obtained at 5 years, 85% contained lamina propria or deeper tissue with complete response demonstrated in 92% of patients, while 8% had focal IM, and there were no buried glands, dysplasia, strictures, or serious adverse events.

Effective BE ablation presumes complete eradication of the abnormal epithelium, inclusive of its stem cells that are believed to accumulate oncogenetic abnormalities that lead to the phenotypic expression of dysplasia and cancer in the epithelial cells [14–17]. Ineffective (incomplete) ablation leaves IM behind and increases the risk for IM to become buried beneath the neosquamous epithelium [18]. This latter phenomenon is also known as subsquamous intestinal metaplasia (SSIM). After the eradication of the BE epithelium, wound healing ensues followed by restoration, in most cases, of a thin nascent squamous epithelium. This neosquamous epithelium thickens over time to a normal stratified squamous epithelium. Several theories exist as to the source of the neosquamous epithelium, including encroachment of adjacent squamous epithelium, extension of cells from the submucosal gland duct lining with conversion to squamous epithelium, and circulating pluripotent stem cells which deposit in the wound and transform to squamous stem cells [19, 20]. Biddlestone et al. have reported that SSIM, when it occurs, resides in the deep portion of the epithelium or in the lamina propria [21]. Adler et al. revealed densely packed Barrett's esophagus glands beneath 300–500 *μ*m of superficial tissue and distorted layered architecture with good histologic correlation at biopsy specimens, using three-dimensional optical coherence tomography [22].

The field of CLE has advanced rapidly with multiple studies published and presented in the past 4 years. Published randomized controlled studies suggest that chromoendoscopy-aided endomicroscopy allows targeting of mucosal biopsy, thereby, increasing the diagnostic yield and decreasing biopsy number. By using the current CLE system, the mucosa can be analyzed at a magnification of about 1000x, but with a maximum penetration depth of the scanning laser light of only 250 *μ*m (too far from the hypothetical residual buried IM), evaluating changes in vessels, connective tissue, and cellular architecture during ongoing endoscopy but without the possibility to diagnose SSIM.

CLE can be performed relatively easily over the esophagus to identify architectural and vascular changes; endomicroscopic changes suggesting the presence of dysplastic or neoplastic changes within BE mucosa include the presence of irregular, black cells with a loss of the normal cellular pattern and distorted subepithelial capillaries with leakage of fluorescein. Since its depth limitation; therefore, we can affirm that CLE is a potentially future diagnostic tool in the surveillance of BE after RFA. Nowadays CLE in Barrett's esophagus after RFA can play an important role in the diagnosis of residual superficial glandular mucosa and/or revealing dysplastic changes of the tissue.

In conclusion residual SSIM after RFA ablation is still not visible, but further refinement in confocal imaging, based on the increase of the penetration depth of the scanning laser light up to 500–600 *μ*m over the lamina propriae and the eventually 3D reconstruction, could increase in the next future the role of CLE in the surveillance of BE.
